# A Wearable Magneto-Inertial System for Gait Analysis (H-Gait): Validation on Normal Weight and Overweight/Obese Young Healthy Adults

**DOI:** 10.3390/s17102406

**Published:** 2017-10-21

**Authors:** Valentina Agostini, Laura Gastaldi, Valeria Rosso, Marco Knaflitz, Shigeru Tadano

**Affiliations:** 1 Department of Electronics and Telecommunications, Politecnico di Torino, Corso Duca degli Abruzzi 24, 10129 Torino, Italy; marco.knaflitz@polito.it; 2 Department of Mechanical and Aerospace Engineering, Politecnico di Torino, Italy, Corso Duca degli Abruzzi 24, 10129 Torino, Italy; laura.gastaldi@polito.it (L.G.); valeria_rosso@polito.it (V.R.); 3 Division of Human Mechanical Systems and Design, Faculty of Engineering, Hokkaido University, Sapporo, Hokkaido 060-8628, Japan; tadano@eng.hokudai.ac.jp

**Keywords:** gait analysis, wearable, STEP32, H-Gait, magneto-inertial sensors, spatio-temporal parameters, joint kinematics, young, overweight, obese

## Abstract

*Background*: Wearable magneto-inertial sensors are being increasingly used to obtain human motion measurements out of the lab, although their performance in applications requiring high accuracy, such as gait analysis, are still a subject of debate. The aim of this work was to validate a gait analysis system (H-Gait) based on magneto-inertial sensors, both in normal weight (NW) and overweight/obese (OW) subjects. The validation is performed against a reference multichannel recording system (STEP32), providing direct measurements of gait timings (through foot-switches) and joint angles in the sagittal plane (through electrogoniometers). *Methods*: Twenty-two young male subjects were recruited for the study (12 NW, 10 OW). After positioning body-fixed sensors of both systems, each subject was asked to walk, at a self-selected speed, over a 14-m straight path for 12 trials. Gait signals were recorded, at the same time, with the two systems. Spatio-temporal parameters, ankle, knee, and hip joint kinematics were extracted analyzing an average of 89 ± 13 gait cycles from each lower limb. Intraclass correlation coefficient and Bland-Altmann plots were used to compare H-Gait and STEP32 measurements. Changes in gait parameters and joint kinematics of OW with respect NW were also evaluated. *Results*: The two systems were highly consistent for cadence, while a lower agreement was found for the other spatio-temporal parameters. Ankle and knee joint kinematics is overall comparable. Joint ROMs values were slightly lower for H-Gait with respect to STEP32 for the ankle (by 1.9° for NW, and 1.6° for OW) and for the knee (by 4.1° for NW, and 1.8° for OW). More evident differences were found for hip joint, with ROMs values higher for H-Gait (by 6.8° for NW, and 9.5° for OW). NW and OW showed significant differences considering STEP32 (*p* = 0.0004), but not H-Gait (*p* = 0.06). In particular, overweight/obese subjects showed a higher cadence (55.0 vs. 52.3 strides/min) and a lower hip ROM (23.0° vs. 27.3°) than normal weight subjects. *Conclusions*: The two systems can be considered interchangeable for what concerns joint kinematics, except for the hip, where discrepancies were evidenced. Differences between normal and overweight/obese subjects were statistically significant using STEP32. The same tendency was observed using H-Gait.

## 1. Background

Gait analysis is used to quantitatively assess normal human locomotion and its changes due to disease and aging, as well as to monitor the effects of therapeutic interventions and rehabilitation [1]. Wearable sensors represent the most recent and advanced technological solution to perform gait analysis since they are less expensive than traditional optoelectronic systems, can be applied outside the laboratory environment in free-living conditions, and can be used in an unrestricted area for long acquisition times [2,3,4,5]. Among wearable sensors, Magnetic and Inertial Measurement Units (MIMUs) are the most promising ones [6,7]. However, since in the clinic high accuracy and repeatability are mandatory, MIMUs’ validity in clinical gait analysis is still an open issue, as documented by many recent studies comparing MIMUs’ performances against a gold standard [6,8,9,10,11].

The main challenge in using MIMUs lies in the fact that position data are not directly measured, but rather obtained by integrating acceleration and angular velocity data [12]. The integration process entails drift errors that affect both spatio-temporal and kinematic data. De-drifting algorithms have been proposed to handle this problem, using the gait cycle features [13,14] or using an additional magnetic sensor and introducing a Kalman filter [15,16] to correct the drift. Even though magnetometers are sensitive to magnetic field distortion [17], it has been shown that sufficient repeatability is obtained along the sagittal plane [18]. Another challenging aspect of the MIMUs is the calibration process, to change from the sensor to the body segment reference system [19,20].

Furthermore, measurements with inertial sensor systems might be affected by soft-tissue artifacts [21,22]. Indeed, any motion measurement method, based on skin-mounted sensors, suffers from artifacts caused by the vibration of the skin and soft tissues beneath it. This problem is already well assessed when using marker-based stereo-photogrammetry [23]. However, a few studies analyzed the influence of soft-tissue artifact when using inertial sensors to perform gait analysis [24]. The problem can be expected to be even more relevant in overweight and obese individuals, where the excess of subcutaneous adipose tissue makes motion analysis measurements particularly challenging. For this reason, when testing a new system, it is not sufficient to consider only young slim participants [23]. Obtaining reliable measurements on these subjects does not guarantee that this result can be extended to the entire population. Hence, it is fundamental to test new systems also in potentially “problematic” conditions. In particular, the usability of wearable sensors should be verified also in overweight and obese subjects. Furthermore, it is particularly important to evaluate changes in gait biomechanics of overweight and obese subjects [25,26], since altered kinematics, in combination with increased joints load, are considered responsible for the development of musculoskeletal pathologies, and particularly knee osteoarthritis [27,28].

H-Gait (Development Code, Laboratory of Biomechanical Design, Hokkaido University, Sapporo, Japan) is a new wearable system dedicated to perform human motion analysis in unconstrained environments. The system comprises MIMU sensors equipped with multidirectional accelerometers, gyroscopes, and magnetometers, as well as custom software routines for computing gait spatio-temporal parameters and joint kinematics, also in 3D. H-Gait was already used to measure gait characteristics both in healthy and pathological subjects [29]. However, a complete validation of the system is still lacking.

In a pilot study [30], the gait parameters obtained with H-Gait were compared to those obtained with a STEP32 (Medical Technology, Torino, Italy) device, a commercial wearable system for gait analysis, used as a reference system. STEP32 directly measures gait data: gait events are detected by foot-switches, while joint angles are measured, in the sagittal plane, by electrogoniometers. This system fully complies with the Medical Device Directive 93/42, was fully characterized from a metrological point of view and holds the CE mark. It is commercially available in Europe and it was already used to study the normal and pathological function of gait, in children, adults and elderly [31,32,33]. However, this system was never used to assess a population of overweight or obese young adults.

Although MIMU sensors have been introduced and validated, also involving joint kinematics in other planes than the sagittal one [19,34], studies on gait involving potentially “problematic” conditions such as overweight/obese populations are still lacking. Therefore, the aim of this work is to validate the H-Gait system against the reference system STEP32, both for normal weight (NW) and overweight/obese (OW) subjects, comparing: a) spatio-temporal parameters, b) ankle, knee, and hip joint kinematics, bilaterally, in the sagittal plane.

## 2. Methods

### 2.1. Participants

Twenty-two male volunteers were enrolled in the study among university students: 12 with normal weight and 10 overweight or obese. Inclusion criteria were: (a) age between 20 and 30 years, (b) body mass index (BMI) between 18.5 and 25 (NW group) or greater than 25 (OW group). Exclusion criteria were any musculoskeletal disorder or neurological condition that could alter gait. Anthropometric data of the participants are reported in Table 1. The differences in age, height, weight and BMI between NW and OW groups were analyzed by means of 2-sample *t*-tests (2-tails, significance level: α = 0.05). This study was approved by the local Institutional Review Board and all procedures conformed to the Helsinki Declaration. Written informed consent was obtained from all participants.

### 2.2. Procedures

The sensors of the two gait analysis systems (H-Gait and STEP32) were positioned, at the same time, on the body of the volunteer.

#### 2.2.1. H-Gait

Seven sensor units (TSDN121, ATR Promotions, Kyoto, Japan) were fixed to the pelvis and the lower limbs of the subject, as it is detailed in the protocol below. Each H-Gait sensor unit (size: 46 mm × 37 mm × 12 mm, weight: 22 g) consists of a tri-axial accelerometer, a tri-axial gyroscope, and a tri-axial magnetometer. The geo-magnetic sensor (AMI306, AICHI STEEL, Tōkai, Japan), accelerometer and gyroscope sensors are incorporated in a MEMS (MPU-6050, InvenSense, Sunnyvale, CA, USA). For each sensor’s component, it is possible to choose a measurement range of interest [14]: accelerometers from 2 to 16 G, gyroscopes from 250 to 2000 dps, magnetometers up to 1200 μT. The sampling rate can be varied from 1 to 1000 Hz.

For this study, the measurement range was set to ± 4 G (accuracy: 0.12 mG) for the accelerometer and ± 500 dps (accuracy: 0.015 dps) for the gyroscope. The accuracy of the magnetometer is 0.3 μT. A sampling rate of 100 Hz was chosen for all sensors. Acquired data were recorded in local data storages (512 Mbyte) and then transferred to a laptop. The H-Gait system was calibrated in order to determine the rotation matrix between each MIMU sensor local coordinate system and the global coordinate system [35].

#### 2.2.2. STEP32

The STEP32 is a multichannel acquisition system for gait analysis, able to record synchronously footswitch signals (for gait event detection), joint kinematics, and a video of the gait test. Six footswitches (size: 10 mm × 10 mm × 0.5 mm, activation force: 3 N) and 6 electrogoniometers (accuracy: 0.5°) were fixed to the lower limbs of the volunteer, to measure the joint kinematics in the sagittal plane. Due to their structure based on an articulated parallelogram, STEP32 electrogoniometers do not require the alignment of the potentiometer shaft with the instantaneous center of rotation of the joint. The STEP32 sampling frequency is equal to 2 kHz. Each sensor was cable-connected to a “patient unit” (size: 60 mm × 30 mm × 150 mm, weight: 300 g) fixed to the subject’s back by means of an elastic belt. Data acquired by the patient unit were then transferred, through a cable, to a PC. 

### 2.3. Protocol

Experiments were conducted indoor in a well-lit room; participants walked barefoot at a self-selected speed, over a straight path of 14 m. A frontal camera, synchronized with the STEP32 system, was mounted on a tripod to video-record the entire gait analysis session. A specific sequence of operations was defined for the calibration procedure and to prepare the subject for the bi-instrumented gait, to optimize the sensors’ placement of both systems. In particular, for the first part of the H-Gait calibration procedure:

(1) Tape measurements between anatomical landmarks were collected from the subject. In particular, the following measurements were taken: pelvis breadth (between the left and right great trochanter), thigh height (between the great trochanter and the lateral epicondyle of the femur), shank height (between the lateral epicondyle of the femur and the lateral malleolus), and sphyrion height (between the lateral malleolus and the floor). Measurements of the lower limbs allow for calculating the body segment coordinate system [36].
(2) Ten reflective markers were placed in correspondence of the volunteer’s greater trochanter, lateral epicondyle of the femur, medial epicondyle of the femur, lateral malleolus and medial malleolus, bilaterally [30].
(3) The volunteer was placed in front of a homogeneous background. Three digital images were taken from the front, left and right sides of the volunteer: the room light was reduced and a flash was used to highlight markers [37]. Subsequently, marker centroids were determined from these images to estimate the inclination of body segments, in order to obtain the rotation matrix to change from the global to the body reference systems (R_GlobalToBody_) [36].
(4) Reflective markers were removed.

The sensors of both systems were then fixed to the subject as follows:

(5) Six STEP32 foot-switches were fixed under both barefoot soles (3 under each foot). In particular, footswitches were positioned beneath the back portion of the heel, the first, and fifth metatarsal heads [38].
(6) Six STEP32 electro-goniometric sensors were fixed in correspondence of ankle, knee and hip joints of each lower limb, with bi-adhesive tape [30].
(7) Elastic bands with Velcro and tape were used to fix the seven H-Gait inertial sensors: two below the medial malleolus [37], two on the shanks in correspondence of the anterior side of the tibia bone, two on the lateral side of the thighs and one on the pelvis, in the posterior center point between the left and right iliac crest. Sensor positions were chosen to minimize motion artifacts and to avoid overlapping among the sensors of the two systems.

**Figure 1:**
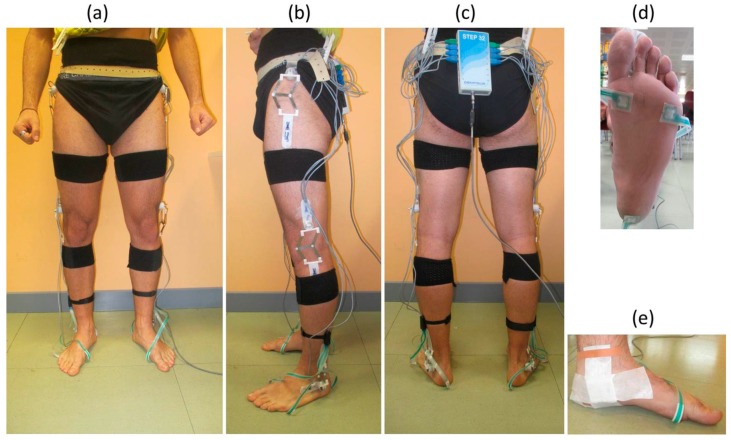
H-Gait and STEP32 sensor positioning. The images show the (**a**) frontal, (**b**) lateral and (**c**) rear view of a subject prepared for the bi-instrumented gait analysis: the MIMU sensor positioned below the medial malleolus is shown in panel (**d**), the footswitches in panel (**e**).

A subject prepared for the bi-instrumented gait is shown in Figure 1. After sensors positioning, the second part of the MIMUs calibration was performed. The subject was asked to assume: a sitting posture and an upright standing posture, for 3 seconds each, to complete H-Gait calibration. Since in the sensor coordinate system the gravitational acceleration vector is different in the two postures, this allows for calculating the axes of the global coordinate system [36]. The rotation matrix to change from the sensors coordinate system to the global coordinate system is then obtained (R_SensorToGlobal_) [36]. Finally, the rotation matrix from the sensor to the body coordinate system is obtained by:

R_SensorToBody _ = R_SensorToGlobal_ R_GlobalToBody_ (1)

The upright standing posture was also used to set the “zeros” of the STEP32 and H-Gait systems to obtain the reference angles for ankle, knee and hip joints. Then, the test started. The subject was asked to perform an initial bilateral knee flexion (approximately 45°), with trunk erect, to synchronize the two systems. Then he was instructed to walk back and forth, along the 14-m straight path, at self-selected speed. Before and after each direction change the subject stopped for 2 seconds. Each walk along the 14-m pathway was considered as a trial. A total of 12 gait trials were acquired for each subject.

## 3. Signal Processing and Data Analysis

For each trial, the average self-selected speed was estimated as the path length (14 m) divided by the time needed to walk through it. Then, the average value over the 12 trials was computed.

### 3.1. H-Gait

The orientation of each sensor in terms of Euler angles was calculated using an extended Kalman filter based on quaternions [36]. By means of the roto-translation matrix defined in Equation (1), it was possible to move from the local frame of each sensor to the anatomical frame of each body segment [36]. Heel contact (HC) and toe off (TO) instants were evaluated through the shank angular velocity, directly measured by MIMU sensors, and the calculated toe trajectory, respectively [30]. HC was used to segment the gait cycles. Then, both HC and TO were used to obtain spatio-temporal parameters. Finally, ankle, knee, and hip joint kinematics, normalized to the gait cycle, was obtained.

### 3.2. STEP32

The STEP32 footswitch signals were debounced, converted to four levels (Heel contact (H), Flat foot contact (F), Push-off (P), Swing (S)), and processed to segment gait cycles [38]. The STEP32 goniometric signals were low-pass filtered (FIR filter, 100 taps, cut-off frequency of 15 Hz) and time-normalized with respect to the gait cycle duration. The following spatio-temporal parameters were measured with both systems: cadence (stride/min), stance (%GC), swing (%GC), and double support (%GC). Joint kinematics of lower limbs, in the sagittal plane, was defined as follow: hip joint angle was the angle between the trunk and the thigh, knee joint angle was the angle between the thigh and the shank, and ankle joint angle was the angle between the shank and the foot. The Range of Motions (ROMs), defined as the difference between the maximum and minimum angles of the kinematic curves, were calculated with both systems. For each subject, spatio-temporal parameters, joint kinematics, and ROMs were measured considering the average over 12 trials. For each parameter, the left and right strides were analyzed separately, except for cadence, for which left and right strides were averaged.

### 3.3. Statistical Analysis

We analyzed if there were any significant difference in the participants’ anthropometric data and in the self-selected gait speed, between NW and OW groups, using a 2-sample *t*-test (2-tails, significance level: α = 0.05).

#### 3.3.1. Agreement between H-Gait and STEP32

For each parameter, the mean difference between H-Gait and STEP32 systems was calculated. The agreement between the H-Gait and STEP32 was analyzed by calculating the intraclass correlation coefficient (ICC) with 95% confidence interval for consistency (2-way mixed) in NW and OW groups [39]. ICC was rated as poor (ICC < 0.40), moderate (0.40 < ICC < 0.60), good (0.60 < ICC < 0.75) or excellent (ICC > 0.75) [39]. However, a high correlation does not necessarily imply that there is good agreement between the systems. For this reason, Bland and Altman plots were also reported. These plots are commonly used to evaluate the agreement among two different instruments or measurement techniques [40]. In the Bland-Altman plots, the mean of the two measurements is assigned as the abscissa (*x*-axis), and their difference as the ordinate (*y*-axis). Along with the scatter plot of between-method differences, the 95% limits of agreement (LoA = 1.96 × SD of between-system difference) are reported [9]. If the differences within mean ± 1.96 SD are not clinically important, the two methods may be used interchangeably.

#### 3.3.2. Comparison between NW and OW Groups

For each acquisition system, a 1-way multivariate analysis of variance (MANOVA) was used to evaluate the differences between NW and OW groups, considering the spatio-temporal parameters and joint ROMs as dependent variables. The average values over NW and OW groups were compared by post-hoc *t*-tests (2-tails, significance level: α = 0.05), when appropriate. The comparison of joint kinematic curves between NW and OW groups were graphically represented for both systems. Statistics were performed with SPSS Software (Version 22, IBM Corporation, Armonk, NY, USA).

## 4. Results

Body mass and BMI were significantly different between NW and OW groups, as expected, while there were no significant differences for age and height (Table 1). The gait speed (1.09 ± 0.12 m/s for NW and 1.05 ± 0.11 m/s for OW) was not significantly different between groups (*p* = 0.5). A total of 42 lower limbs were analyzed: 22 for NW and 20 for OW. An average of 89 ± 13 gait cycles was considered for each lower limb, which was sufficient to obtain good reliability of joint angles and to overcome intrinsic variability [41].

### 4.1. Agreement between H-Gait and STEP32

**Figure 2:**
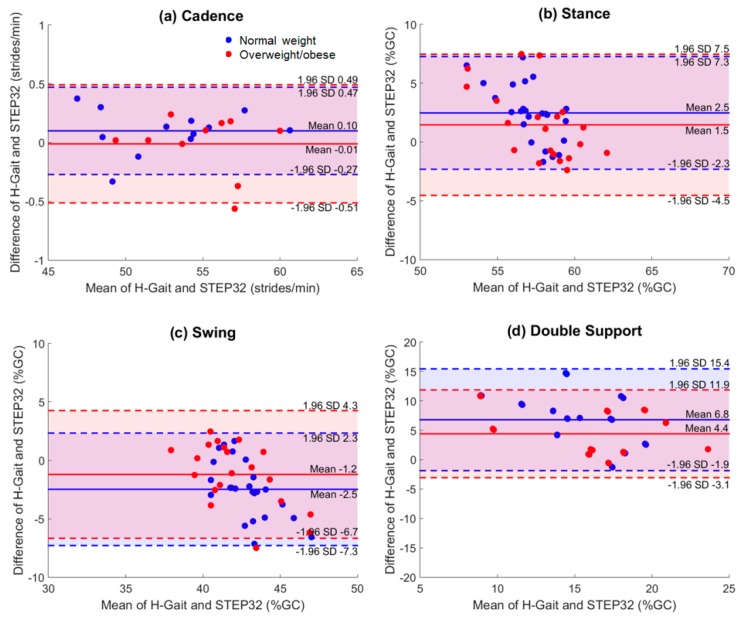
Bland-Altman plots: spatiotemporal parameters. Bland-Altman plots are shown for (**a**) cadence; (**b**) stance; (**c**) swing and (**d**) double support, for normal weigh subjects (blue dots) and overweight/obese subjects (red dots). In each plot, the mean and LoA (mean ± 1.96 SD) are shown for both groups. Values from left and right lower limbs were represented as separate dots, except for cadence, where left/right values were averaged.

**Figure 3:**
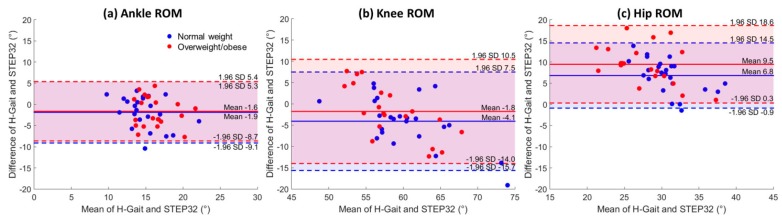
Bland-Altman plots: joint ROMs. Bland-Altman plots are shown for (**a**) ankle ROM; (**b**) knee ROM and (**c**) hip ROM; for normal weigh subjects (blue dots) and overweight/obese subjects (red dots). In each plot, the mean and LoA (mean ± 1.96 SD) are shown for both groups. Values from left and right lower limbs were represented as separate dots.

For each spatio-temporal parameter and joint ROM, the mean difference, ICC, and ICC-confidence intervals between H-Gait and STEP32 systems are reported in Table 2. Furthermore, spatio-temporal parameters and joint ROMs are graphically represented with Bland-Altman plots in Figure 2 and Figure 3, respectively.

For what concerns spatio-temporal parameters, the differences between the systems was almost zero for cadence (Table 2). In contrast, H-Gait showed higher values compared to STEP32 for stance and double support, and lower values for swing, in both groups (Table 2). Overall, smaller differences between the systems could be observed in the OW than in the NW group. The ICC agreement between systems was excellent for cadence in both groups; it was also excellent for double support and good for stance and swing, but only in the OW group. In all the other cases, the ICC agreement was moderate or poor.

For what concerns joint ROMs, small differences were observed for the ankle and knee (H-Gait values lower than STEP32 ones), while more evident differences were found for hip joint (H-Gait values higher than STEP32 ones). The ICC agreement between systems was moderate for ankle ROM, in both groups. It was also moderate for knee ROM in OW group. In all the other cases, the ICC agreement was good.

### 4.2. Comparison between NW and OW Groups

**Figure 4:**
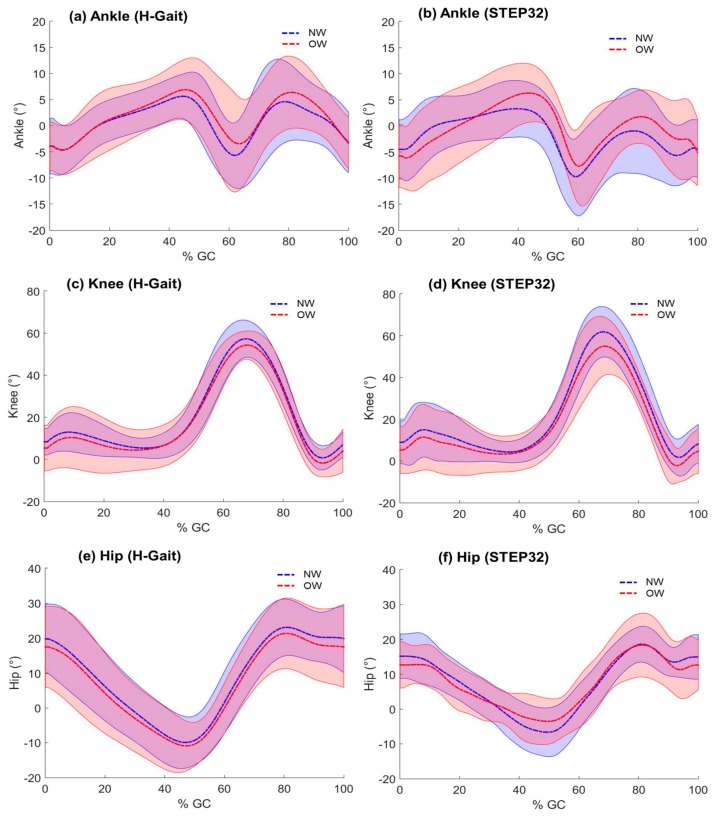
Joint kinematics. Ankle, knee, and hip kinematics are compared between normal weight (NW) and overweight/obese subjects (OW), both for H-Gait and STEP32 systems. Mean ± SD is reported.

For each spatio-temporal parameter and joint ROM, average values for the NW and the OW group for both systems are reported in Table 3. The *p*-values of post-hoc *t*-tests (2-tails, significance level: α = 0.05) were reported when appropriate. Furthermore, joint kinematic curves for the two BMI groups and both systems are graphically compared in Figure 4.

The NW and OW groups were significantly different considering STEP32 measurements (MANOVA: *p* = 0.0004), while they were just above the limit of significance considering H-Gait measurements (*p* = 0.06). Cadence was slightly augmented and hip ROM was reduced in OW with respect to NW group.

## 5. Discussion

In this study we compared the performances of two wearable systems for gait analysis: H-gait and STEP32, with the latest used as reference. Contextually, we compared the gait of normal and overweight/obese healthy subjects, analyzed with both systems. Therefore, the discussion focuses first on the comparison between the systems and, then, between NW and OW groups.

### 5.1. Agreement between H-Gait and STEP32

#### 5.1.1. Spatio-Temporal Parameters

The two systems were highly consistent for cadence, while a lower agreement was found for the other spatio-temporal parameters (Table 2, Figure 2). Overall, we found a shortened stance and prolonged swing using STEP32 compared to H-Gait. Indeed, for what concern the forefoot, STEP32 switches were positioned below metatarsal heads, and not below the hallux. As a consequence, the meaning of the terms “stance” and “swing” for STEP32 may differ from their strict definition [42], giving an error in the duration of the stance/swing phases. The difference between systems is even greater for the double support parameter. In fact, in this case, the difference due to a shortened stance is added to the difference due to a prolonged swing.

We observed that the differences found in spatio-temporal parameters, due to foot-switch positioning in STEP32, had a greater impact on NW with respect to OW subjects. In literature, it is recognized that overweight and obesity affect the biomechanical function of the foot [43]. Significantly higher pressures are found under the metatarsal region in obese subjects compared to normal weight individuals and this difference is consistent with a greater foot-contact area in the heavier subjects [44]. Hence, we expect that OW subjects have a reduced forefoot rocker compared to NW subjects, with ground reaction forces applied to metatarsal heads rather than toes, during foot off. Consequently, the foot switch position (under the metatarsal heads instead of hallux) probably introduces a smaller error on the toe off measurements, in the OW compared to NW group. This could explain the lower discrepancy observed between the toe off instant measured by foot switches, and the one measured by accelerometers, in OW subjects.

#### 5.1.2. Joint Kinematics

Some studies have validated inertial sensor systems for what concerns spatio-temporal parameters [6] and the joint kinematics [19,34]. However, to the best of our knowledge, such a validation was not provided for potentially “problematic” conditions such as overweight and obese subjects. In this study, we compared the joint kinematics curves obtained with H-Gait and STEP32 on normal and overweight subjects. Overall, ankle, knee, and hip curves obtained with the two systems showed similar morphologies, both for NW and OW group (Figure 4). The dispersion of the hip joint curves was slightly smaller using STEP32 system with respect to H-Gait. It can be observed a profile with bumps in STEP32 curves of OW subjects, especially in the hip and, to a lesser extent, in the knee kinematics. This can be most probably related to soft tissue artifacts that are expected to be more pronounced in OW subjects. In contrast, soft tissue artifacts were not evident in H-Gait curves, probably due to the different sensors positioning and the fixation with elastic bands.

Differences in ROMs were evaluated and graphically represented with Bland-Altman plots. For the ankle and knee joints only small ROM differences were observed, with H-Gait showing slightly lower values with respect to STEP32. In constrast, a more evident difference was found for hip joint, with H-Gait showing an increased ROM with respect to STEP32. The hip ROM measured by H-gait seems to be closer to the values reported in the literature [26,45], although there are a few studies on the hip kinematics of young healthy subjects and they were collected for different purposes and with different protocols. Hence, it is difficult to compare our results with other works.

To explain the hip ROM discrepancy between systems, the presence of kinematic crosstalk might be hypothesized [46]. In literature, kinematic crosstalk is a phenomenon in which movements performed in one plane are recorded as a false signal in the orthogonal planes. Any technique that measures joint kinematics is potentially subject to crosstalk error. In particular, flexible electrogoniometers are known to be affected by it [47]. However, STEP32 electrogoniometers are based on articulated parallelograms, which do not require a perfect alignment of the potentiometer shaft with the instantaneous center of rotation of the joint. Hence, the kinematic crosstalk is expected to be minimized using STEP32 electrogoniometers. Although there are no studies investigating kinematic crosstalk in inertial sensor systems, also H-Gait might be affected by this phenomenon. In particular, kinematic crosstalk could be due to the MIMU attached to the pelvis, which rotates mostly around the vertical axis. This kind of movement may be not well compensated by the Kalman filter, which can introduce artifacts at the yaw pelvis angle. Errors caused by these artifacts can propagate to hip flexion/extension angles. For these reasons, it is difficult to establish if the hip ROM is overestimated by H-Gait or, on the contrary, it is underestimated by STEP32.

### 5.2. Comparison between NW and OW Groups

Although the main focus of the paper was the comparison between H-Gait and STEP32 systems, biomechanical gait changes were evaluated for OW compared to NW subjects. In fact, the knowledge of gait parameters of obese subjects is still relatively poor, particularly in young adults. Within the limited literature, preferred walking speeds have been consistently reported to be slower in the obese when compared with normal-weight individuals [48]. Consequently, it is not clear whether the altered sagittal kinematics noted in obese adults represents a unique gait characteristic in this population or merely the adoption of a slower walking speed [48], since it is well established that the walking speed influences gait parameters [49]. In our study, the self-selected speed was not significantly different in overweight/obese subjects with respect to normal weight ones. This may help the direct comparison of gait biomechanics between groups. Overall, the differences in gait parameters observed between OW and NW groups were more evident when considering STEP32 measurements with respect to H-Gait ones. For this reason, in the rest of this section, we refer to the STEP32 system only.

Although several studies reported no differences between obese and normal-weight subjects on cadence [27], we found that cadence was higher in OW with respect to NW subjects. We also found a reduced hip ROM in OW with respect to NW subjects (Table 3). This is confirmed by previous works on obese subjects. De Vita et al. [50] suggested that when walking slower or at the same velocity, obese individuals use a more erect walking pattern, with less hip and knee flexion and more ankle plantarflexion compared to lean individuals. Furthermore, the reduced hip ROM observed in OW subjects is consistent with the reduced step length and larger step width reported in obese gait [51].

### 5.3. Limitations

Although range of motion is an important kinematic index for comparing the performance of the two methods (H-Gait and STEP32), other indexes to evaluate waveform similarity could have been introduced. Among others, it is important to mention the Linear Fit Method [52], the Mean Absolute Variability [53] and the Waveform Distortion [15]. These methods might be considered in future studies.

## 6. Conclusions

This study demonstrates the usability of a wearable system for gait analysis based on magneto-inertial sensors (H-Gait), not only on normal weight, but also on overweight/obese subjects, that are known to be a more critical population. In particular, this work provides a first attempt to evaluate the accuracy of the reconstructed joint kinematics using magneto-inertial sensors against a reference system (STEP32). In fact, while this evaluation was already reported in literature for spatio-temporal parameters, to the best of our knowledge, no other works validated joint kinematic measurements.

Overall, the two systems can be considered interchangeable for what concerns joint kinematics, except for the hip, where discrepancies were evidenced. Comparing subjects with different body mass index, differences between normal and overweight/obese subjects were statistically significant using STEP32 measurements. The same tendency was observed considering H-Gait measurements, but the difference between groups was just above the limit of significance.

## Figures and Tables

**Table 1:** Anthropometric data: normal weight (NW) and overweight/obese (OW) subjects.

	Age (years)	Height (cm)	Body Mass (kg)	BMI
NW (N = 12)	26.2 ± 1.5	180.4 ± 8.8	74.3 ± 8.2 *	22.8 ± 1.1 *
OW (N = 10)	25.6 ± 2.2	175.3 ± 7.6	95.5 ± 12.4 *	31.1 ± 3.3 *

Values are mean ± standard deviation over the population. Significant differences between normal weight and overweight/obese subjects are indicated with * (*p* < 0.0001).

**Table 2:** Between-methods consistency: mean difference between H-Gait and STEP32 measurements, intraclass correlation coefficient (ICC), and ICC 95%-confidence intervals (CI). For cadence, left and right strides were averaged, while for the other parameters they were treated as independent samples.

	NW			OW		
	Difference ^a^ (Mean ± SD)	ICC ^b^	ICC 95%-CI	Difference ^a^ (Mean ± SD)	ICC ^b^	ICC 95%-CI
*Spatio-Temporal Parameters*						
Cadence (strides/min)	0.1 ± 0.2	0.99	[0.97, 1.0]	−0.01 ± 0.3	1.00	[1.00, 1.00]
Stance (%GC)	2.5 ± 2.5	0.23	[−0.78, 0.67]	1.5 ± 3.0	0.66	[0.14, 0.87]
Swing (%GC)	−2.5 ± 2.5	0.36	[−0.47, 0.73]	−1.2 ± 2.8	0.67	[0.16, 0.87]
Double support (%GC)	6.8 ± 4.4	0.44	[−0.30, 0.76]	4.4 ± 3.8	0.82	[0.53, 0.93]
*Joint Kinematic Parameters*						
Ankle ROM (°)	−1.9 ± 3.7	0.49	[−0.18, 0.78]	−1.6 ± 3.6	0.43	[−0.44, 0.77]
Knee ROM (°)	−4.1 ± 5.9	0.72	[0.36, 0.88]	−1.8 ± 6.2	0.56	[−0.13, 0.82]
Hip ROM (°)	6.8 ± 3.9	0.61	[0.11, 0.83]	9.5 ± 4.7	0.69	[0.21, 0.88]

**^a^** Difference between H-Gait and STEP32 measurements (mean value ± standard deviation). **^b^** Intraclass correlation coefficient: poor (ICC < 0.40); moderate (0.40 < ICC < 0.60); good (0.60 < ICC < 0.75); excellent (ICC > 0.75).

**Table 3:** Comparison between normal-weight (NW) and overweight/obese (OW) subjects for spatio-temporal and joint kinematics parameters measured using H-Gait and STEP32, respectively.

	H-Gait			STEP32		
	NW	OW	*p*-value	NW	OW	*p*-value
*Spatio-Temporal Parameters*						
Cadence (strides/min)	52.9 ± 4.0	55.0 ± 3.0	-	52.6 ± 4.1*	55.0 ± 3.1*	0.04
Stance (%GC)	58.0 ± 2.2	58.7 ± 2.0	-	55.9 ± 2.7	57.2 ± 3.6	0.18
Swing (%GC)	41.8 ± 1.5	41.5 ± 2.0	-	44.2 ± 2.7	42.7 ± 3.4	0.12
Double support (%GC)	18.6 ± 2.6	19.0 ± 4.2	-	11.8 ± 4.6	14.5 ± 5.4	0.08
*Joint Kinematic Parameters*						
Ankle ROM (°)	13.9 ±2.5	15.3 ± 2.8	-	15.8 ± 3.7	16.9 ± 3.2	0.30
Knee ROM (°)	58.4 ± 4.4	57.5 ± 3.1	-	62.5 ± 7.8	59.2 ± 7.3	0.17
Hip ROM (°)	34.1 ± 2.6	32.5 ± 3.8	-	27.3 ± 4.6 *	23.0 ± 5.6 *	0.01

Values are reported as mean ± standard deviation over the population. The post-hoc *p*-values are reported for STEP32 (MANOVA *p* = 0.0004), but not for H-Gait (MANOVA *p* > 0.05). Significant differences between NW and OW are indicated with * (*p* < 0.05).

## Abbreviations

BMI	Body Mass Index
MIMUs	Magnetic and Inertial Measurement Units
NW	Normal Weight
OW	Overweight
ROM	Range of Motion
